# Preoperative Left Ventricular Global Longitudinal Strain Identifies Aortic Stenosis Patients with Improved Postoperative Recovery of Left Ventricular Geometry: A Prospective Cohort Study

**DOI:** 10.21470/1678-9741-2020-0529

**Published:** 2022

**Authors:** Planinka Zafirovska, Rodney Alexander Rosalia, Ljubica Georgievska Ismail, Niki Matveeva, Zan Mitrev

**Affiliations:** 1 Zan Mitrev Clinic, Skopje, the Republic of North Macedonia.; 2 University Clinic of Cardiology, Skopje, the Republic of North Macedonia.; 3 Institute of Anatomy, University of Cyril and Methodius, Skopje, the Republic of North Macedonia.

**Keywords:** Aortic Valve Stenosis, Heart Ventricules, Ventricular Remodeling, Severity of Illness Index, Biomarkers, Echocardoography, Clinical Decision-Making

## Abstract

**Introduction:**

The left ventricular ejection fraction (LVEF) is commonly used as a marker of aortic stenosis (AS) disease severity and to indicate surgical intervention. However, an LVEF <50% identifies mainly advanced disease. Hence, earlier detection of subclinical LV systolic dysfunction may improve clinical decision-making. The global longitudinal strain (GLS) can identify subclinical systolic dysfunction at earlier stages of AS progression even in the presence of preserved LVEF. To this end, we evaluated the preoperative prognostic significance of the LVGLS to identify patients who will undergo a more extensive postoperative LV reverse remodeling as a surrogate marker for clinical recovery.

**Methods:**

We performed a prospective observational study based on detailed pre- and postoperative 2D transthoracic echocardiographic examinations, including strain analysis with speckle tracking. We screened 60 consecutive patients with severe AS and a preoperative LVEF ≥50% indicated for surgery; 39 patients met the study entry criteria and consented to their participation.

**Results:**

The median age was 67 (range 30-79) years; 56.4% were female. At baseline, the GLS was 61.64±7.22%. Surgery led to an improvement in the GLS; the mean difference was 3.23% [95% CI=1.96 to 4.49%] during a median follow up time of 5 (interquartile range 4-6) months. The preoperative GLS correlated with the postoperative LV mass index (LVMI) r=0.526, *P*=0.001 and the intraventricular septal thickness in diastole (IVSd) r=0.462, *P*=0.003. Furthermore, patients with a normal GLS (≤-18.9%) at baseline experienced a better recovery of their LV morphology and systolic function during the postoperative course compared to those with an abnormal GLS (>-18.9%). The effect size, hedges g, was at least >0.75 for the LVMI, IVSd, intraventricular septal thickness in systole (IVSs), left ventricular posterior wall thickness in diastole (LVPWd) and LVEF, suggesting a clinically significant difference between subgroups at follow-up.

**Conclusion:**

A normal preoperative left ventricular global longitudinal strain is associated with an improved left ventricular reverse remodeling and systolic function following surgery to resolve aortic stenosis.

**Table:** Abbreviations, acronyms & symbols

ACE	= Angiotensin-converting enzyme
ARB	= Angiotensin receptor blockers
ASA	= Acetylsalicylic acid
AVAI	= Aortic valve area index
BB	= Beta-blockers
BMI	= Body mass index
BSA	= Body surface area
CABG	= Coronary artery bypass grafting
CAD	= Coronary artery disease
CCB	= Calcium channel blockers
CCS	= Canadian Cardiovascular Society
ECG	= Electrocardiogram
ESC	= European Society of Cardiology
GLS	= Global longitudinal strain
IVSd	= Intraventricular septal thickness in diastole
IVSs	= Intraventricular septal thickness in systole
LVEDd	= Left ventricular end-diastolic dimension
LVEF	= Left ventricular ejection fraction
LVGLS	= Left ventricular global longitudinal strain
LVH	= Left ventricular hypertrophy
LVMI	= Left ventricular mass index
LVPWd	= Left ventricular posterior wall thickness in diastole
LVRR	= Left ventricular reverse remodeling
NYHA	= New York Heart Association
RWT	= Relative wall thickness

## INTRODUCTION

The assessment of left ventricular (LV) systolic function is crucial in the risk stratification of aortic stenosis (AS) patients^[1]^. Current guidelines recognise a ejection fraction (EF) <50% as a class I indication for aortic valve replacement (AVR)^[2]^. Nevertheless, clinical decision-making, according to the left ventricular ejection fraction (LVEF), has been questioned in recent years^[3]^. LVEF may remain normal for years, due to compensatory mechanisms, despite the occurrence of deep sub-clinical structural and functional myocardial changes that dictate disease progression, which can affect the clinical outcome^[4]^.

It is well established that patients with severe AS and significant LV systolic dysfunction can benefit from surgical aortic valve replacement (SAVR). Nevertheless, severe AS patients with LV systolic dysfunction are at higher risk to experience postoperative complications with an increased risk of intrahospital and long-term mortality in comparison to patients with preserved LV function.

The global longitudinal strain (GLS) is a more accurate marker of myocardial fibrosis compared to LVEF. In comparison to other indexes of LV systolic function, the parameters of LV longitudinal deformation are superior in detecting myocardial dysfunction and damage. Current evidence suggests that patients with severe AS have sub-clinical LV systolic dysfunction, resulting in distorted GLS values despite preserved LVEF^[5]^. We speculate that the normalisation of GLS values might signify a reversal of pathological myocardial changes and correlate with left ventricular reverse remodeling (LVRR). GLS can be routinely evaluated using 2D-speckle tracking echocardiography.

Consequently, GLS has the potential to be incorporated into clinical decision-making in patients with severe AS and to predict SAVR procedural success according to the postoperative LVRR.

This study evaluated the correlation between preoperative GLS and postoperative echocardiography parameters associated with LVRR. Furthermore, we analysed whether GLS can stratify patients who will experience more pronounced resolution of LV hypertrophy and improvement in systolic function following SAVR.

## METHODS

**Fig. 1:**
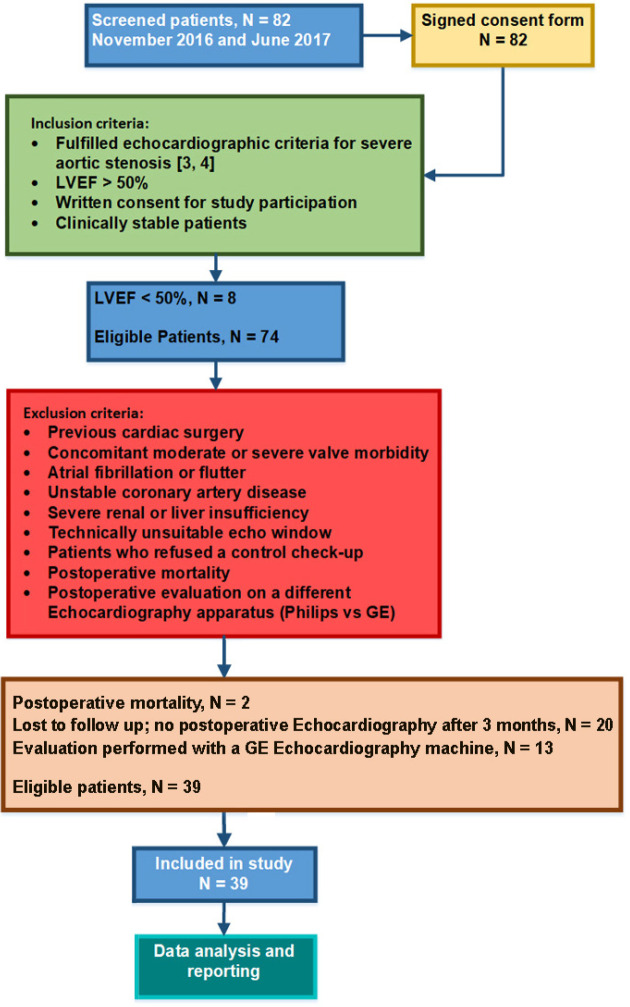
STROBE study flow diagram. Patient eligibility evaluation, inclusion and exclusion criteria and patient eligibility.

We prospectively screened 82 consecutive patients indicated for SAVR at our clinic between November 2016 and June 2017. The STROBE study flow diagram describing the inclusion and exclusion criteria is shown in Figure 1.

Inclusion criteria were:

- Fulfilled echocardiographic criteria for severe aortic stenosis.
- LVEF >50%.
- Written consent for study participation.
- Clinically stable patients.

Exclusion criteria were:

- Previous cardiac surgery.
- Concomitant moderate or severe valve morbidity.
- Atrial fibrillation or flutter.
- Unstable coronary artery disease.
- Severe renal or liver failure.
- Technically unsuitable echo window.
- Patients who refused a control check-up.
- Postoperative mortality.
- Postoperative evaluation on a different echocardiography device (Philips *vs.* GE).

The final study cohort consisted of n=39 patients. All patients provided a signed informed consent form to participate in this observational study. The Zan Mitrev Clinic’s Ethics Committee approved the study.

We performed a medical history evaluation, physical examination, biochemical analysis and electrocardiography (ECG) and a coronary angiogram for the possibility of existing CAD.

Echocardiographic examinations were performed on a Philips Epiq 7 Cardiology Ultrasound Machine; saved recordings were examined using the Philips IntelliSpace Cardiovascular Portal platform.

We performed the measurements according to the guidelines and recommendations^[2]^.

EF was calculated by Simpson’s method, mean and peak trans-aortic gradients were determined using the continuous wave Doppler method, and aortic valve area was calculated with the continuity equation^[6]^.

Tissue Doppler imaging was used to obtain peak systolic (S’) and peak early diastolic (E’) mitral annular velocities measured on the septal and lateral sides of the mitral annulus and after the calculation of E/E’^[7]^. Relative wall thickness (RWT) was calculated according to the following formula 2 × PWd/LVEDd: “double the thickness of the posterior wall divided by left ventricular end-diastolic dimension”^[8]^.

The LV mass was calculated according to the linear method using the American Society of Echocardiography formula: $\text{LV mass }=0.8\times (1.04\left[(LVIDd+\text{ PWTd }+\text{ SWTd }{)}^{3}-(LVIDd{)}^{3}\right)+0.6g$^[9]^,

Where:

LVIDd = Left ventricular internal diameter at end-diastole

PWTd = Posterior wall thickness in diastole

SWTd = Septal wall thickness in diastole and indexed to the body surface area (m^2^/kg).

Mitral annular plane of systolic excursion (MAPSE) was measured in millimetres with M-mode echocardiography at four different points (septal, lateral, anterior and inferior) in apical 4- and 2-chamber views; a value ≥10 mm was considered within the reference range^[10]^.

High-quality ECG-gated images were obtained and recorded with frame rate >50 frames/s in long apical axis 4- and 2- chamber views; recordings were subsequently analysed using 2-D speckle tracking, where segmental strains were presented as a bull’s-eye map. A GLS value of -18.9% was set as the cut-off for normal longitudinal strain as recommended by the vendor^[9]^.

### Ethics Approval and Consent To Participate

The Zan Mitrev Clinic’s ethics committee approved the clinical practice and treatment procedures described in this case series.

### Consent For Publication

The Zan Mitrev Clinic’s ethics committee approved the publication of clinical data under the condition of full anonymity.

### Availability of Data and Material

All data generated or analysed during this study are included in this published article and its supplementary information files.

### Surgical Technique

Surgical aortic valve repair proceeds according to the standard protocol for cardiopulmonary bypass (CPB) placing patients under mild hypothermic conditions (32°C). Myocardial protection is achieved through continuous retrograde and antegrade cardiac perfusion with warm blood cardioplegia.

### Statistical Analysis

Categorical parameters were summarised as absolute numbers and percentages. Continuous data are shown as mean±SD or median+interquartile range (IQR).

Continuous variables were evaluated using the D’Agostino-Pearson normality test - independent parametric data were analysed using the Student’s *t*-test, and non-parametric continuous variables were assessed using the Mann-Whitney test for independent comparisons. Comparisons of preoperative *versus* postoperative data were performed using a paired T-test or Wilcoxon signed-rank test for non-parametric data.

Fisher’s exact test was applied to evaluate the association between categorical variables with the outcome. Regression analysis between preoperative GLS and echocardiographic markers were performed using Pearson’s or Spearman’s correlation testing. We adopted the hedges’ *g* as a measure for the effect sizes due to unequal and small samples sizes.

Data were analysed with the statistical softwares Graphpad Prism, version 7.03 and Statsdirect, version 3.1.20.

## RESULTS

### Patient Characteristics and Procedure Overview

Baseline patient characteristics are presented in Table 1. All patients had symptomatic aortic valve stenosis with clinical indication for SAVR according to European Society of Cardiology guidelines: AS severity based on aortic valve area index (AVAI), mean pressure gradient and peak jet velocity. Hypertension was the most frequent comorbidity (84.6% of patients). In 9 cases (23.1%), SAVR was combined with coronary artery bypass grafting (CABG) surgery. No cases of operative mortality were observed during a follow-up period of 219 patient-months. No patients were lost to follow-up.

**Table 1:** Basic characteristics of 39 patients with severe aortic stenosis undergoing surgical treatment.

Median age in years (range)	67 (30-79)
Gender, N (%)	
Male	17 (43.6)
Female	22 (56.4%)
BMI (kg/m^2^)	28.5±4.1
Hypertension, N (%)	33 (84.6%)
Dyslipidemia, N (%)	19 (48.7)
Type II diabetes mellitus, N (%)	9 (23.1)
NYHA, N (%)	
II	30 (76.9)
III	7 (17.9)
IV	1 (0.03)
CCS, N (%)	
0	30 (76.9)
I	1 (0.03)
II	7 (17.9)
III	1 (0.03)
Mild aortic regurgitation, N (%)	12 (30.8%)
Mild mitral regurgitation, N (%)	9 (23.1%)
Medications, N (%)	
ACE inhibitors	32 (94.9)
ARB	5 (12.9)
BB	32 (94.9)
CCB	7 (17.9)
Diuretics	7 (17.9)
ASA	35 (89.7)
Statins	37 (94.9)
CABG	9 (23.1)
Carotid artery disease, N (%)	3 (7.7)
Type of implanted valve, N (%)	
Bioprosthetic	18 (46.2)
Mechanical	6 (15.4)
Reconstruction	15 (38.5)

ACE=angiotensin-converting enzyme; ARB=angiotensin receptor blockers; ASA=acetylsalicylic acid; BB=beta-blockers; BMI=body mass index; CABG=coronary artery bypass grafting; CAD=coronary artery disease; CCB=calcium channel blockers; CCS=Canadian Cardiovascular Society; ECG=electrocardiogram; LVH=left ventricular hypertrophy; NYHA=New York Heart Association

The echocardiographic parameters before and after SAVR are summarised in Supplemental Tables 1 to 4. All patients had severe AS with preserved LVEF. The mean (± SD) preoperative AVAI, transvalvular mean pressure gradient (T_MG_), transvalvular peak aortic jet velocity (pT_AS_) and LVEF were 0.42±0.09 cm^2^/m^2^, 37.7±15.1 mmHg and 4.08±0.81 m/s and 61.64±7.22%, respectively.

The mean (± SD) GLS was -16.58±4.25%, and the LV fractional shortening was 36.95±9.92% before surgical intervention.

We observed LV hypertrophy in all patients. The mean (± SD) left ventricular mass index (LVMI) in males was 216.3±48.45 g/m^2^, of which 15 out of 17 had concentric hypertrophy. In female patients, the mean (± SD) LVMI was 150.3±47.39 g/m^2^ with 19 out of 22 having concentric hypertrophy at baseline.

After a median follow-up time of 5 (IQR 4-6) months, several parameters associated with AS severity were improved (Supplemental Tables 1 to 4). SAVR resulted in notable improvements in overall LV dimensions and LV systolic function. Furthermore, a significant reduction of E/e’ values and increase in average MAPSE (Supplemental Table 2) point to improved diastolic and systolic function after SAVR.

**Fig. 2:**
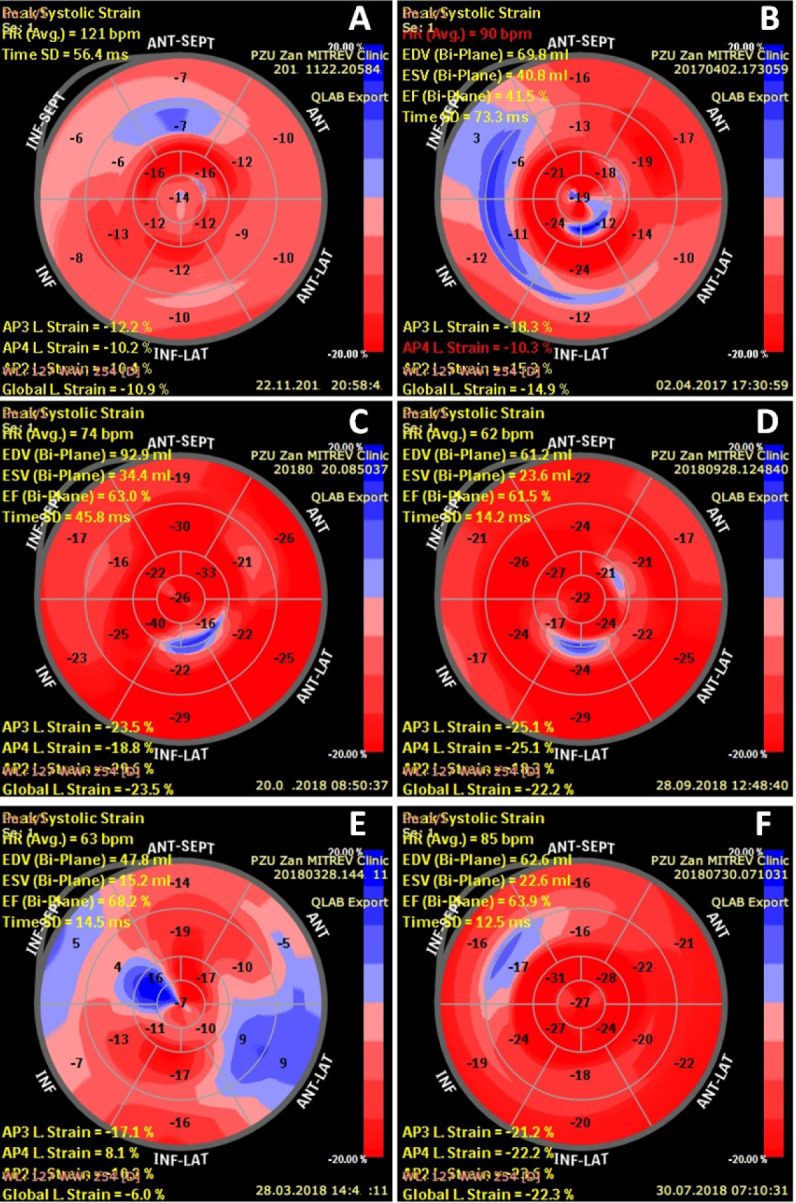
Representative bull’s-eye images of patients presenting with normal versus abnormal preoperative GLS. Panels A and B show preoperative and postoperative bull’s-eye images of a patient with preoperative abnormal GLS who experienced minor, but clinically insufficient, improvement in LV systolic function and myocardial contractility. Panels C and D show perioperative bull’s-eye images of a patient with normal GLS. Panels E and F display images of a patient with impaired subclinical LV systolic function at baseline which improved during the postoperative course.

**Fig. 3:**
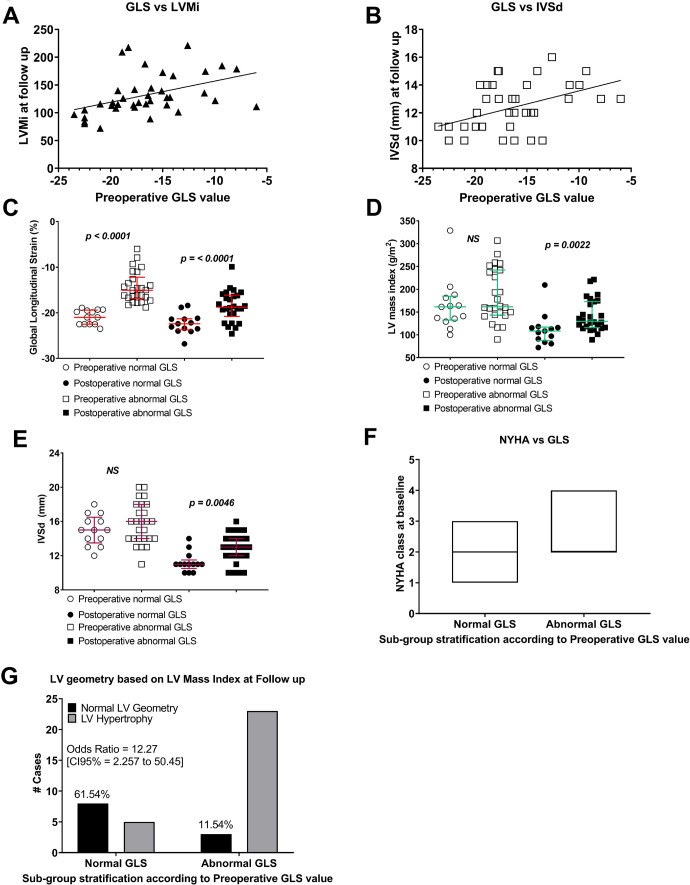
Overview of echocardiography correlated with the GLS. Linear regression line represents the correlation between the GLS and A) left ventricular mass index and B) intraventricular thickness at diastole. Correlation coefficients are embedded in the graphs. C) Scatter plots depict preoperative (open symbols) and postoperative (closed symbols) data of the global longitudinal strain based sub-groups, stratified according to a “normal GLS” (≤-18.9%) cohort (n=13) (circles) and “abnormal GLS” (>-18.9%) cohort (n=26) (squares), D) left ventricular mass index and E) intraventricular septal thickness at diastole. F) Scatter plots show the preoperative left ventricular mass index according to the NYHA class. G) Contingency graph presents the percentage of patients who experienced a full recovery of LV geometry within a median follow-up period of 5 (IQR 4-6 months). The odds ratio is embedded in the graphs as a measure of effect size.

Finally, we observed a gradual normalisation of LV strains compared to baseline values, in particular, the GLS, which improved by 3.23% [1.96 to 4.49%], *P*=0.0001 (Supplemental Table 4; Figures 2 and 3).

### Left Ventricular Global Longitudinal Strain as a pre-SAVR predictive marker for Left Ventricular Mass Regression

We determined the association between preoperative LVGLS and various other echocardiography parameters linked to LVRR at follow-up.

**Supplemental Fig. 1:**
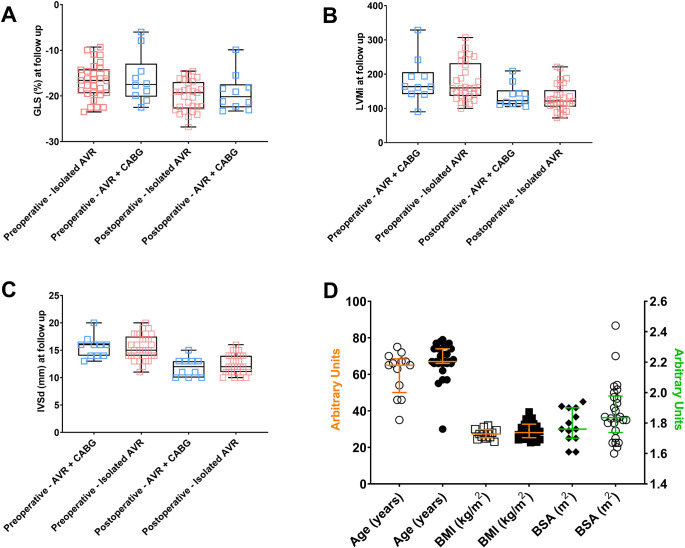
Normal vs. abnormal GLS age, BMI and BSA comparative analysis and evaluation of postoperative left ventricular geometry of patients undergoing isolated SAVR versus patients undergoing SAVR and CABG. A) The global longitudinal strain based sub-groups, stratified according to a “normal GLS” (≤-18.9%) cohort (n=13) (circles) and “abnormal GLS” (>-18.9%) cohort (n=26) (squares) compared based on age, BMI and BSA. Evaluation of the preoperative and postoperative B) global longitudinal strain, C) left ventricular mass index and D) intraventricular septal thickness at diastole.

**Supplemental Figs. 2 to 3:**
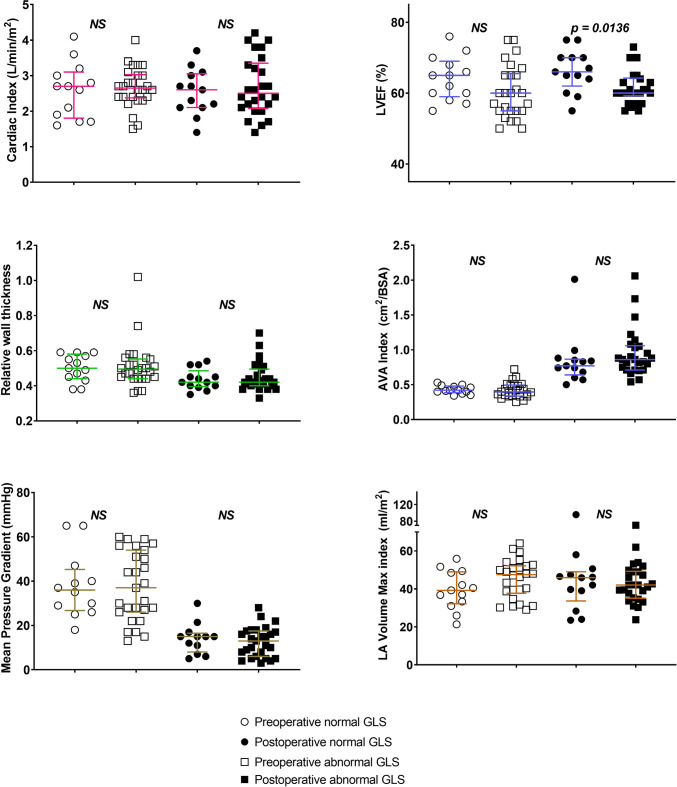

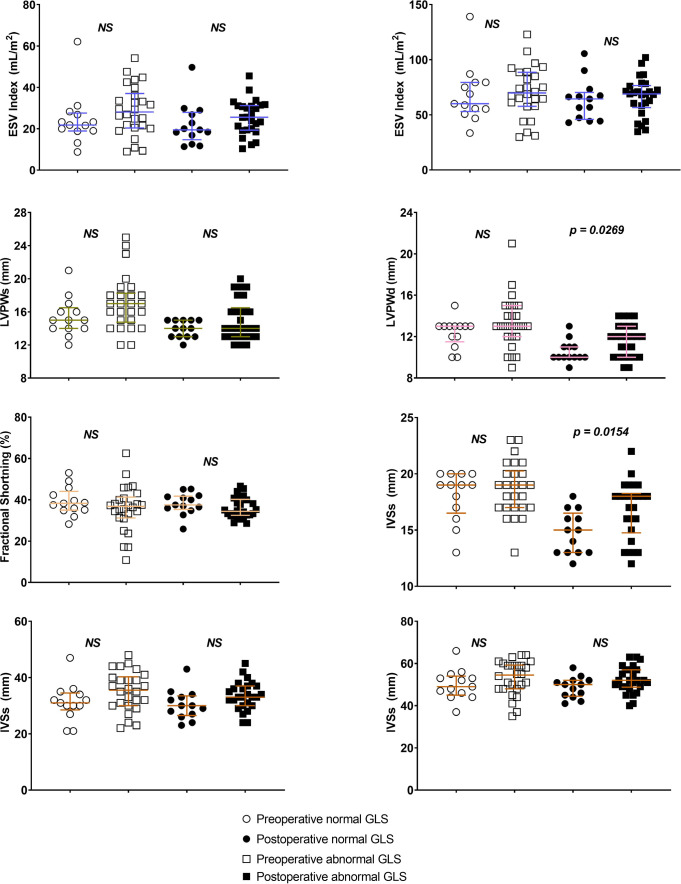
Overview of echocardiography correlated with the left ventricular global longitudinal strain. Scatter plots depict the preoperative (open symbols) and postoperative (closed symbols) data of the global longitudinal strain based sub-groups, stratified according to a “normal GLS” (≤-18.9%) cohort (n=13) (circles) and “abnormal GLS” (>-18.9%) cohort (n=26) (squares).

Preoperative GLS values showed significant correlations with several markers associated with LVRR, such as LVEF, LVPW, interventricular septal thickness and LVMI (Table 2). The biggest effect size was observed between preoperative GLS and post-operative IVSd (r_p_=0.462; *P*=0.0031) and LVMI (r_s_=0.562; *P*<0.0001) (Figures 3C to E and Table 3; Supplemental Figures 1 and 2).

**Table 2:** Correlation between preoperative LVGLS and LVRR echocardiographic parameters.

	Correlation coefficient*	β	*P*-value
GLS	R_P_ 0.507	0.4153	0.0010
IVSd	R_P_ 0.462	0.1886	0.0031
IVSs	R_P_ 0.429	0.2549	00.0064
LVPWd	R_s_ 0.356	0.1055	0.0263
LVPWs	R_P_ 0.343	0.1732	0.0323
LVEF	R_P_ -0.326	-0.4272	0.0430
LVMI^#^	R_s_ 0.526	3.810	0.0006

* Only echo parameters with a regression coefficient >0.3 are shown.

# LVM was excluded from analysis in favour of the BSA corrected value, the LVMI.

GLS=global longitudinal strain; IVSd=intraventricular septal thickness in diastole; IVSs=intraventricular septal thickness in systole; LVEF=left ventricular ejection fraction; LVPWd=left ventricular posterior wall thickness in diastole; LVPWs=left ventricular posterior wall thickness in systole; LVMI=left ventricular mass index; R_P_=Pearson’s correlation coefficient; R_S_=Spearman’s rank correlation coefficient

**Table 3:** Echocardiography parameters associated with LVRR. Postoperative comparison of normal GLS (n=13) *versus* abnormal GLS (n=26) subgroups.*

Parameter	Mean difference + [95% CI]	Effect size hedges g	*P*-value
GLS (%)	-3.825 [1.756 to 5.893]	1.27	0.0061^1^
AVAI	0.1050 [-0.1407 to 0.3507]	0.29	0.3920^1^
CI (L/min/m^2^)	0.1500 [-0.3754 to 0.6754]	0.20	0.5664^1^
LVEF (%)	-4.577 [-8.155 to -0.9985]	0.88	0.0136^1^
LVFS (%)	-2.113 [-5.644 to 1.419]	0.41	0.2331^1^
IVSd (mm)	1.654 [0.5747 to 2.733]	1.05	0.0036^1^
IVSs (mm)	2.038 [0.4131 to 3.664]	0.86	0.0154^1^
LVEDv index (ml/m^2^)	2.837 [-9.411 to 15.09]	0.16	0.6414^1^
LVESv index (ml/m^2^)	3.492 [-2.728 to 9.712]	0.39	0.2626^1^
LVEDd (mm)	3.577 [-0.4584 to 7.612]	0.61	0.0807^1^
LVESd (mm)	3.192 [-0.3377 to 6.722]	0.62	0.075^1^
LVPWd (mm)	1.115 [0.1347 to 2.096]	0.77	0.0269^1^
LVPWs (mm)	1.077 [-0.3762 to 2.530]	0.51	0.1417^1^
RWT	0.01462 [-0.03801 to 0.06724]	0.18	0.5770^1^
LV mass index (g/m^2^)	21.25 [-9.300 to 53.40]	0.88	0.0022^2^
LA volume index (ml/m^2^)	-2.1 [-11.32 to 7.119]	0.16	0.6471^1^

* Postoperative comparison between sub-groups, “normal GLS” *versus* “abnormal GLS”, are based on a preoperative GLS cut-off value of -18.9%.

1 Student’s t-test.

2 Mann-Whitney test.

AVAI=aortic valve area index; CI=cardiac index; GLS=global longitudinal strain; IVSd=intraventricular septal thickness in diastole; IVSs=intraventricular septal thickness in systole; LA=left atrium; LV=left ventricle; LVEF=left ventricular ejection fraction; LVEDd=left ventricular end-diastolic dimension; LVEDv=left ventricular end-diastolic volume; LVESv=left ventricular end systolic volume; LVPWd=left ventricular posterior wall thickness in diastole; LVPWs=left ventricular posterior wall thickness in systole; LVMI=left ventricular mass index; RWT=relative wall thickness

Next, we examined the prognostic significance of GLS. For this purpose, the cohort was stratified into two sub-groups according to the recommendations of the vendor Philips Epiq 7 for a GLS cut-off value of -18.9%; “normal” = GLS ≤-18.9% group (n=13) and the “abnormal”= GLS >-18.9% group, n=26. The sub-groups were equally matched for age, BMI and BSA (Supplemental Figure 3D).

At baseline, both sub-groups had similar LV morphology and systolic functions, except significant differences in GLS (Figures 3C to E). We observed a trend suggesting higher NYHA status in the abnormal GLS sub-group. However, this difference did not reach statistical significance. On the other hand, NYHA class was associated (r_p_=0.477; *P*=0.0023) with the extent of preoperative LV hypertrophy; patients with NYHA class 3 had a more pronounced LV hypertrophy compared to those with NYHA class 2, mean difference in LVMI was 59.71 g/m^2^ (95% CI 17.21 to 102.4 g/m^2^), *P*=0.0072 (Figure 3F).

All patients successfully underwent SAVR and experienced an uncomplicated postoperative course. Patients who required concomitant CABG experienced a similar postoperative LVRR as those who underwent isolated SAVR (Supplemental Figures 1B to D)

The clinical condition of the whole cohort improved at follow-up; notably, the “normal GLS” subgroup (≤-18.9%) had a more favourable LVMI compared to the “abnormal” subgroup (>-18.9%) (Figure 3B). Furthermore, the normal GLS sub-group were more likely to recover a healthy LVMI compared to those with an abnormal GLS, OR 12.27 (95% CI 2.257 to 50.45), P=0.0021 (Figure 3G).

Finally, we evaluated the postoperative values of candidate echocardiography outcome measures associated with LVRR (Table 3). The most prominent effects between the “normal” and “abnormal” subgroups at follow-up were observed for markers of LV geometry.

In conclusion, patients with a normal GLS experience a faster normalisation of LV dimensions after SAVR.

## DISCUSSION

Aortic stenosis (AS) is the most common degenerative valvular heart disease; a result of immune-mediated calcification of the valve leaflets. Failure of compensatory mechanisms over time leads to clinical symptoms as a consequence of progressive fibrosis, impairment of myocardial contractility and reduced LVEF^[1]^. However, reduced LVEF (<50%) manifests in the late stages of the disease and has been associated with irreversible myocardial dysfunction and is an independent risk factor for sudden death in patients with severe AS.

To this end, more sensitive markers for the early stages of AS that overcome the limitations of LVEF-guided clinical decision-making are warranted^[11]^. Early detection of subclinical systolic dysfunction may optimise the timing of aortic valve intervention^[12]^. GLS is a promising and sensitive marker of the subclinical systolic dysfunction^[13,14]^.

In the present study, we assessed the prognostic significance of LVGLS in a cohort of severe AS patients with preserved LVEF. We demonstrate that 1) the baseline GLS value strongly correlates with several echocardiography parameters linked to LV hypertrophy and LV systolic functions. Moreover, 2) patients with a normal GLS (<-18.9%) experienced a more pronounced LVRR during the early postoperative period. In contrast, LVRR extent was significantly impaired in patients with an abnormal GLS (≥-18.9%) at baseline.

Notably, sub-groups were matched for age, gender and BMI; both groups were similar concerning the degree of AS severity, LV hypertrophy, LVEF and NYHA class at baseline. The main difference between sub-groups before SAVR was their sub-clinical LV systolic function as determined by speckle-tracking echocardiography strain analysis (Figure 3C and Supplemental Figure 1A).

SAVR forms the cornerstone of AS management. It has been shown that the degree of LV hypertrophy and extent of LVRR after SAVR determines long-term clinical prognosis^[15]^.

Even though the evaluation of GLS can detect subclinical systolic dysfunction with high sensitivity^[13,14]^, strain analysis by speckle tracking echocardiography is currently not implemented in major clinical practice guidelines^[2]^. Nonetheless, our data suggest that the preoperative GLS has the potential to classify patients who will experience accelerated LVRR after SAVR. Thus, a normal GLS is associated with better postoperative recovery of normal LV geometry.

Our data align with recent reports that advocate the inclusion of LVGLS analysis to improve risk stratification of patients with severe AS, facilitate clinical decision-making and timing of aortic valve replacement^[16]^. Of importance, the LVGLS is independently associated with all-cause mortality in AS patients^[17]^.

Heterogeneity in GLS measurements among published clinical studies hampers the inclusion of the LVGLS analysis in clinical practice guidelines; the recorded measurements vary among different vendors because of proprietary differences in the software used to calculate deformation^[18]^. Reported reference values of GLS vary from -15.9% to -22.1%^[19]^. Nevertheless, our comparative study was built on a cut-off value of -18.9% as recommended when using the Philips Epiq 7^[9]^.

Accordingly, patients with a normal GLS (<-18.9%) experienced a faster recovery of normal LV geometry and substantial improvement in their LV functionality (Supplemental Table 6). In contrast, abnormal GLS values at baseline translated to a modest LVRR, characterised by a slower normalisation of their LV geometry during the follow-up period.

Based on our data, one could hypothesise that normal GLS values pertain to LV cardiomyocytes with the intrinsic capacity to overcome interstitial fibrosis^[1]^ and attenuate signalling effectors underlying pathologic remodelling.

Resolution of pathological LV hypertrophy is generally considered a marker of favourable prognosis and prolonged survival^[18]^; on the other hand, recent reports suggest that a rapid improvement of the LVMI is paradoxically linked to a high(er) 30-day mortality^[19,20]^. There were no early mortality cases in our study; currently, our long-term follow-up is still ongoing, and long-term mortality can not be assessed yet. Given the complexities of LV mass regression post-AVR^[21]^, the limitations of 2D imaging-derived calculations^[19]^, SV adaptions^[21]^ it will be of high interest to determine potential differences in mid-to-long-term mortality rates between the “normal” and “abnormal” GLS subgroups.

Our study has several limitations that require further discussion; first, our results pertain to a single-centre, observational retrospective study. A more controlled prospective setting, *e.g*. stratification based on NYHA class, classification of AS stages^[22]^ and strict comparison based on comorbidities such as coronary artery disease, might strengthen the data. Also, a subgroup analysis comparing patients with or without concomitant CABG revealed no differences in the extent of LVRR based on postoperative values of GLS, LVMI and IVSd (Supplemental Figure 1).

Second, the study was powered to detect an effect size of >0.5. Consequently, the sample size for the sub-group comparisons was too small to confirm other trends with a smaller effect size; for instance, the association between the preoperative NYHA class and GLS value.

Third, the subgroups should be followed over a more extended period to confirm a possible association between the postoperative LVRR with adverse cardiovascular events and (all-cause) mortality.

Despite these limitations, our conclusions are based on highly significant mean/median differences (Supplemental Tables 1 to 4, correlation coefficients (Supplemental Table 5) and large effect sizes, >0.75 (Supplemental Table 6), which combined may be interpreted as clinically relevant observations.

## CONCLUSION

In conclusion, a normal preoperative LVGLS is associated with improved postoperative recovery of normal LV geometry and function. GLS correlates with several echocardiography markers of LVRR.

The results advocate the use of the LVGLS as an early and more sensitive marker to establish AS stenosis severity, facilitate indication for AVR and classify patients with a better postoperative prognosis following surgery.

**Table:** Authors' roles & responsibilities

PZ	Substantial contributions to the conception or design of the work; or the acquisition, analysis or interpretation of data for the work; drafting the work or revising it critically for important intellectual content; final approval of the version to be published
RAR	Substantial contributions to the conception or design of the work; or the acquisition, analysis or interpretation of data for the work; final approval of the version to be published
LGI	Substantial contributions to the conception or design of the work; or the acquisition, analysis or interpretation of data for the work; drafting the work or revising it critically for important intellectual content; final approval of the version to be published
NM	Substantial contributions to the conception or design of the work; or the acquisition, analysis or interpretation of data for the work; drafting the work or revising it critically for important intellectual content; final approval of the version to be published
ZM	Substantial contributions to the conception or design of the work; or the acquisition, analysis or interpretation of data for the work; final approval of the version to be published

## Figures and Tables

**Supplemental Table 1:** Echocardiographic assessment of aortic valve stenosis severity.

Parameter	Preoperative (mean±SD)	Postoperative (mean±SD)	Mean difference + [95%CI]	*P*-value
AV V_max_ (m/s)	4.08±0.81	2.26±0.68	1.816667 [1.526954 to 2.10638]	0.0001
AV mean PG (mmHg)	37.7±15.1	13.1±6.71	24.669231 [20.277947 to 29.060514]	0.0001
AVA (cm^2^)	0.7±0.16	1.71±0.64	-0.939231 [-1.140747 to-0.737715]	0.0001
AVAi (cm^2^/m^2^)	0.42±0.09	0.92±0.36	-0.494142 [-0.605465 to-0.382819]	0.0001
Velocity ratio	0.24±0.10	0.48±0.18	-0.234282 [-0.299867 to-0.168697]	0.0001

AVA=aortic valve area; AV V_max_=aortic valve maximal jet velocity; PG mean=aortic transvalvular mean pressure gradient

**Supplemental Table 2:** Comparison of echocardiographic parameters of LV systolic function.

Parameter	Preoperative (mean±SD)	Postoperative (mean±SD)	Mean difference + [95%CI]	*P*-value
LVEDd (mm)	52.03±7.88	51.08±6.03	0.948718 [-1.117519 to 3.014955]	0.3585
LVESd (mm)	33.87±7.06	32.44±5.29	1.435897 [-0.385427 to 3.257222]	0.1188
IVSd (mm)	15.51±2.20	12.33±1.74	3.179487 [2.470562 to 3.888413]	0.0001
IVSs (mm)	18.49±2.30	16.13±2.53	2.358974 [1.532562 to 3.185387]	0.0001
PWd (mm)	13.00±2.21	11.28±1.50	1.717949 [1.156735 to 2.279162]	0.0001
PWs (mm)	16.51±3.07	14.64±2.14	1.871795 [1.105482 to 2.638108]	0.0001
LVEDVi (mL/m^2^)	70.06±23.81	65.43±17.61	4.630762 [-1.992858 to 11.254383]	0.1651
LVESVi (mL/m^2^)	27.35±12.23	24.39±9.07	2.966168 [-0.283569 to 6.215905]	0.0724
LVEF (%)	61.64±7.22	63.18±5.57	-1.538462 [-3.76931 to 0.692387]	0.1708
LVFS (%)	36.95±9.92	36.67±5.16	0.276951 [-2.382619 to 2.93652]	0.8342
SV index (mL/m^2^)	37.24±9.26	38.19±10.80	-0.949589 [-5.000994 to 3.101816]	0.6379
CI (L/min/m^2^)	2.63±0.63	2.63±0.76	0.00076 [-0.28403 to 0.285551]	0.9957
MAPSEa (mL/m^2^)	10.38±2.37	13.28±2.75	-2.898974 [-3.615348 to-2.182601]	0.0001
LVMi (g/m^2^)	216.3±48.45	147.7±37.03	68.646338 [49.519472 to 87.773204]	0.0001
LVMi (g/m^2^)	150.3±47.39	119.79±33.81	30.419468 [15.18258 to 45.65635]	0.0005
RWT	0.51±0.11	0.45±0.08	0.06221 [0.026731 to 0.09769]	0.0001

CI=cardiac index/cardiac output indexed to BSA; IVSd=intraventricular septal thickness in diastole; IVSs=intraventricular septal thickness in systole; LVEDd=left ventricular end-diastolic dimension; LVESd=left ventricular end-systolic dimension; PWd=posterior wall thickness in diastole; PWs=posterior wall thickness in systole; LVEF=left ventricular ejection fraction; LVESdVi=left ventricular end-systolic dimension volume indexed to BSA; LVESVi=left ventricular end-systolic volume; LVFS=left ventricular fractional shortening; MAPSEa=mitral annular plane systolic excursion; LVMi=left ventricular mass indexed to BSA; RWT=relative wall thickness; SVi=systolic volume indexed by BSA

**Suplemental Table 3:** Comparison of echocardiographic parameters of LA volumes and function, right heart and diastolic LV function.

Parameter	Preoperative (mean±SD)	Postoperative (mean±SD)	Pre- *vs*. postoperative mean difference + [95%CI]	*P*-value
LAVi (mL/m^2^)	43.34±10.36	43.73±13.26	-0.394292 [-4.270921 to 3.482336]	0.838
E/A ratio	0.88±0.37	1.02±0.33	-0.138378 [-0.273611 to-0.003146]	0.0452
DT (ms)	221.24±62.98	195.02±57.48	26.216216 [2.954565 to 49.477867]	0.0283
IVRT (ms)	78.96±28.99	79.00±21.78	-0.521739 [-12.568526 to 11.525048]	0.9292
e’ septal (cm/s)	5.62±1.12	6.69±1.51	-1.147222 [-1.661105 to-0.633339]	0.0001
e’ lateral (cm/s)	6.42±1.50	7.99±1.88	-3.113889 [-4.257644 to-1.970134]	0.0001
e’ average (cm/s)	6.03±1.20	7.39±1.46	2.823908 [1.043912 to 4.603903]	0.0028
E/e’ septal	5.61±2.31	6.75±2.40	0.69729 [-1.142512 to 2.537093]	0.4468
E/e’ lateral	6.18±2.47	9.34±2.43	-3.11389 [-4.257644 to-1.970134]	0.0008
E/e’ average	5.93±2.26	8.13±1.92	2.823908 [1.043912 to 4.603903]	0.0028
RA (mm)	35.41±5.42	35.56±5.61	-0.153846 [-1.827602 to 1.51991]	0.8554
RV (mm)	32.51±5.75	33.38±5.55	0.128205 [-1.430296 to 1.686706]	0.8686
TAPSE (mm)	18.36±3.61	17.13±3.09	1.230769 [-0.064732 to 2.52627]	0.062
PAPs (mmHg)	26.31±9.30	25.97±9.73	0.333333 [-2.57305 to 3.239717]	0.8176

LAVi=maximum left atrial volume indexed to BSA; DT=deceleration time; E velocity=early mitral inflow velocity; e’ velocity=early diastolic mitral annular tissue Doppler velocity; IVRT=isovolumetric relaxation time; LAi=left atrial diameter indexed to BSA; PAPs=systolic pulmonary artery pressure; RA=right atrial diameter; RV=right ventricular diameter; TAPSE=tricuspid annular plane systolic excursion

**Supplemental Table 4:** Comparison of LV longitudinal strain before and after SAVR.

Parameter	Preoperative (mean±SD)	Postoperative (mean±SD)	Pre- *vs* postoperative mean difference + [95%CI]	*P*-value
LV LS LAX (%)	-17.82±5.29	-19.50±7.91	1.682051 [-1.008301 to 4.372403]	0.2133
LV LS 4c (%)	-15.71±5.49	-24.52±28.28	8.810256 [-0.226499 to 17.847012]	0.0557
LV LS 2c (%)	-16.42±4.50	-19.18±3.84	2.758974 [1.596198 to 3.921751]	0.0001
GLS (%)	-16.58±4.25	-19.81±3.48	3.228205 [1.964529 to 4.491881]	0.0001

4c=4-chamber view; 2c=2-chamber view; GLS=global LV longitudinal strain; LAX=long axis; LV=left ventricle; LS=longitudinal strain

**Supplemental Table 5:** Echocardiographic parameters associated with left ventricular reverse remodeling. Postoperative comparison of normal GLS (N=13) versus abnormal GLS (N=26) subgroups.

Parameter	Mean difference + [95%CI]	Effect size Hedges g	*P*-value
GLS (%)	-3.825 [1.756 to 5.893]	1.27	0.0061^1^
AVA index	0.1050 [-0.1407 to 0.3507]	0.29	0.3920^1^
CI (L/min/m^2^)	0.1500 [-0.3754 to 0.6754]	0.20	0.5664^1^
LVEF (%)	-4.577 [-8.155 to-0.9985]	0.88	0.0136^1^
LVFS (%)	-2.113 [-5.644 to 1.419]	0.41	0.2331^1^
IVSd (mm)	1.654 [0.5747 to 2.733]	1.05	0.0036^1^
IVSs (mm)	2.038 [0.4131 to 3.664]	0.86	0.0154^1^
LVEDv index (ml/m^2^)	2.837 [-9.411 to 15.09]	0.16	0.6414^1^
LVESv index (ml/m^2^)	3.492 [-2.728 to 9.712]	0.39	0.2626^1^
LVEDd (mm)	3.577 [-0.4584 to 7.612]	0.61	0.0807^1^
LVESd (mm)	3.192 [-0.3377 to 6.722]	0.62	0.075^1^
LVPWd (mm)	1.115 [0.1347 to 2.096]	0.77	0.0269^1^
LVPWs (mm)	1.077 [-0.3762 to 2.530]	0.51	0.1417^1^
RWT	0.01462 [-0.03801 to 0.06724]	0.18	0.5770^1^
LV mass index (g/m^2^)	21.25 [-9.300 to 53.40]	0.88	0.0022^2^
LA volume index (ml/m^2^)	-2.1 [-11.32 to 7.119]	0.16	0.6471^1^

* Postoperative comparison between sub-groups, “normal GLS” versus “abnormal GLS”, are based on a preoperative GLS cut-off value of-18.9%

1 Student’s t-test.

2 Mann-Whitney test. AVA=aortic valve area; CI=cardiac index; GLS=global LV longitudinal strain; IVSd=intraventricular septal thickness in diastole; IVSs=intraventricular septal thickness in systole; LA=left atrium; LV=left ventricle; LVEDd=left ventricular end-diastolic dimension; LVESd=left ventricular end-systolic dimension; LVEDv=left ventricular end-diastolic volume; LVESv=left ventricular end-systolic volume; LVPWd=left ventricular posterior wall thickness in diastole; LVPWs=left ventricular posterior wall thickness in systole; RWT=relative wall thickness

**Supplemental Table 6:** Correlation between preoperative GLS and echocardiographic parameters of LVRR.

	Correlation coefficient	β	*P*-value
GLS	R_P_ 0.507	0.4153	0.0010
IVSd	R_P_ 0.462	0.1886	0.0031
IVSs	R_P_ 0.429	0.2549	00.0064
LVPWd	R_s_ 0.356	0.1055	0.0263
LVPWs	R_P_ 0.343	0.1732	0.0323
LVEF	R_P_-0.326	-0.4272	0.0430
LVMi^#^	R_s_ 0.526	3.810	0.0006

* Only echo parameters with a regression coefficient >0.3 are shown. R_P_=Pearson’s correlation coefficient. R_S_=Spearman’s rank correlation coefficient.

# LVM was excluded from analysis in favour of the BSA corrected value, LVMi. GLS=global LV longitudinal strain; IVSd=intraventricular septal thickness in diastole; IVSs=intraventricular septal thickness in systole; LVEF=left ventricular ejection fraction; LVMi=left ventricular mass index; LVPWd=left ventricular posterior wall thickness in diastole; LVPWs=left ventricular posterior wall thickness in systole

## References

[1] Lindman BR, Clavel MA, Mathieu P, Iung B, Lancellotti P, Otto CM (2016). Calcific aortic stenosis. Nat Rev Dis Primers.

[2] Baumgartner H, Falk V, Bax JJ, De Bonis M, Hamm C, Holm PJ (2018). 2017 ESC/EACTS guidelines for the management of valvular heart disease. Rev Esp Cardiol (Engl Ed).

[3] Everett RJ, Clavel MA, Pibarot P, Dweck MR (2018). Timing of intervention in aortic stenosis: a review of current and future strategies. Heart.

[4] Elmariah S (2015). Patterns of left ventricular remodeling in aortic stenosis: therapeutic implications. Curr Treat Options Cardiovasc Med.

[5] Potter E, Marwick TH (2018). Assessment of left ventricular function by echocardiography: the case for routinely adding global longitudinal strain to ejection fraction. JACC Cardiovasc Imaging.

[6] Baumgartner H, Hung J, Bermejo J, Chambers JB, Edvardsen T, Goldstein S (2017). Recommendations on the echocardiographic assessment of aortic valve stenosis: a focused update from the European association of cardiovascular imaging and the American society of echocardiography. J Am Soc Echocardiogr.

[7] Kadappu KK, Thomas L (2015). Tissue Doppler imaging in echocardiography: value and limitations. Heart Lung Circ.

[8] Devereux RB, Alonso DR, Lutas EM, Gottlieb GJ, Campo E, Sachs I (1986). Echocardiographic assessment of left ventricular hypertrophy: comparison to necropsy findings. Am J Cardiol.

[9] Lang RM, Badano LP, Mor-Avi V, Afilalo J, Armstrong A, Ernande L (2015). Recommendations for cardiac chamber quantification by echocardiography in adults: an update from the American society of echocardiography and the European association of cardiovascular imaging. Eur Heart J Cardiovasc Imaging.

[10] Luszczak J, Olszowska M, Drapisz S, Plazak W, Kaznica-Wiatr M, Karch I (2013). Assessment of left ventricle function in aortic stenosis: mitral annular plane systolic excursion is not inferior to speckle tracking echocardiography derived global longitudinal peak strain. Cardiovasc Ultrasound.

[11] Badiani S, van Zalen J, Treibel TA, Bhattacharyya S, Moon JC, Lloyd G (2016). Aortic stenosis, a left ventricular disease: insights from advanced imaging. Curr Cardiol Rep.

[12] Vollema EM, Sugimoto T, Shen M, Tastet L, Ng ACT, Abou R (2018). Association of left ventricular global longitudinal strain with asymptomatic severe aortic stenosis: natural course and prognostic value. JAMA Cardiol.

[13] Shi J, Pan C, Kong D, Cheng L, Shu X (2016). Left ventricular longitudinal and circumferential layer-specific myocardial strains and their determinants in healthy subjects. Echocardiography.

[14] Gavina C, Falcão-Pires I, Pinho P, Manso MC, Gonçalves A, Rocha-Gonçalves F (2016). Relevance of residual left ventricular hypertrophy after surgery for isolated aortic stenosis. Eur J Cardiothorac Surg.

[15] Ng ACT, Prihadi EA, Antoni ML, Bertini M, Ewe SH, Ajmone Marsan N (2018). Left ventricular global longitudinal strain is predictive of all-cause mortality independent of aortic stenosis severity and ejection fraction. Eur Heart J Cardiovasc Imaging.

[16] Shah AM, Solomon SD (2012). Myocardial deformation imaging: current status and future directions. Circulation.

[17] Yingchoncharoen T, Agarwal S, Popović ZB, Marwick TH (2013). Normal ranges of left ventricular strain: a meta-analysis. J Am Soc Echocardiogr.

[18] Kalam K, Otahal P, Marwick TH (2014). Prognostic implications of global LV dysfunction: a systematic review and meta-analysis of global longitudinal strain and ejection fraction. Heart.

[19] Pibarot P, Borger MA (2017). The left ventricular mass regression paradox following surgical valve replacement: a real phenomenon or a mathematical glitch?. Structural Heart.

[20] Kadkhodayan A, Lin G, Popma JJ, Reardon MJ, Little SH, Adams DH (2017). A paradox between LV mass regression and hemodynamic improvement after surgical and transcatheter aortic valve replacement. Structural Heart.

[21] Rusinaru D, Bohbot Y, Ringle A, Maréchaux S, Diouf M, Tribouilloy C (2018). Impact of low stroke volume on mortality in patients with severe aortic stenosis and preserved left ventricular ejection fraction. Eur Heart J.

[22] Généreux P, Pibarot P, Redfors B, Mack MJ, Makkar RR, Jaber WA (2017). Staging classification of aortic stenosis based on the extent of cardiac damage. Eur Heart J.

